# Cyclosporiasis Surveillance — United States, 2011–2015

**DOI:** 10.15585/mmwr.ss6803a1

**Published:** 2019-04-19

**Authors:** Shannon M. Casillas, Rebecca L. Hall, Barbara L. Herwaldt

**Affiliations:** 1 Division of Parasitic Diseases and Malaria, Center for Global Health, CDC

## Abstract

**Problem/Condition:**

Cyclosporiasis is an intestinal illness caused by the parasite *Cyclospora cayetanensis*, which is transmissible by ingestion of fecally contaminated food or water. Cyclosporiasis is most common in tropical and subtropical regions of the world. In the United States, foodborne outbreaks of cyclosporiasis have been linked to various types of imported fresh produce (e.g., basil, raspberries, and snow peas). Validated molecular typing tools, which could facilitate detection and investigation of outbreaks, are not yet available for *C. cayetanensis*.

**Period Covered:**

2011–2015.

**Description of System:**

CDC has been conducting national surveillance for cyclosporiasis since it became a nationally notifiable disease in January 1999. As of 2015, cyclosporiasis was a reportable condition in 42 states, the District of Columbia, and New York City (NYC). Health departments voluntarily notify CDC of cases of cyclosporiasis through the National Notifiable Diseases Surveillance System and submit additional case information using the CDC cyclosporiasis case report form or the Cyclosporiasis National Hypothesis Generating Questionnaire (CNHGQ).

**Results:**

For the 2011–2015 surveillance period, CDC was notified by 37 states and NYC of 2,207 cases of cyclosporiasis, including 1,988 confirmed cases (90.1%) and 219 probable cases (9.9%). The annual number of reported cases ranged from 130 in 2012 to 798 in 2013; the annual population-adjusted incidence rate ranged from 0.05 cases per 100,000 persons in 2012 to 0.29 in 2013. A total of 415 patients (18.8%) had a documented history of international travel during the 14 days before illness onset, 1,384 (62.7%) did not have a history of international travel, and 408 (18.5%) had an unknown travel history. Among the 1,359 domestically acquired cases with available information about illness onset, 1,263 (92.9%) occurred among persons who became ill during May–August. During 2011–2015, a total of 10 outbreaks of cyclosporiasis associated with 438 reported cases were investigated; a median of 21 cases were reported per outbreak (range: eight to 162). A food vehicle of infection (i.e., a food item or ingredient thereof) was identified (or suspected) for at least five of the 10 outbreaks; the food vehicles included a berry salad (one outbreak), cilantro imported from Mexico (at least three outbreaks), and a prepackaged salad mix from Mexico (one outbreak).

**Interpretation:**

Cyclosporiasis continues to be a U.S. public health concern, with seasonal increases in reported cases during spring and summer months. The majority of cases reported for this 5-year surveillance period occurred among persons without a history of international travel who became ill during May–August. Many of the seemingly sporadic domestically acquired cases might have been associated with identified or unidentified outbreaks; however, those potential associations were not detected using the available epidemiologic information. Prevention of cases and outbreaks of cyclosporiasis in the United States depends on outbreak detection and investigation, including identification of food vehicles of infection and their sources, which could be facilitated by the availability of validated molecular typing tools.

**Public Health Action:**

Surveillance for cases of cyclosporiasis and efforts to develop and validate molecular typing tools should remain U.S. public health priorities. During periods and seasons when increased numbers of domestically acquired cases are reported, the CNHGQ should be used to facilitate outbreak detection and hypothesis generation. Travelers to areas of known endemicity (e.g., in the tropics and subtropics) should follow food and water precautions similar to those for other enteric pathogens but should be advised that use of routine chemical disinfection or sanitizing methods is unlikely to kill *C. cayetanensis*. Health care providers should consider the possibility of *Cyclospora* infection in persons with persistent or remitting-relapsing diarrheal illness, especially for persons with a history of travel to areas of known endemicity or with symptom onset during spring or summer. If indicated, laboratory testing for *Cyclospora* should be explicitly requested because such testing is not typically part of routine examinations for ova and parasites and is not included in all gastrointestinal polymerase chain reaction panels. Newly identified cases of cyclosporiasis should be promptly reported to state or local public health authorities, who are encouraged to notify CDC of the cases.

## Introduction

Cyclosporiasis is an intestinal illness caused by the unicellular, coccidian parasite *Cyclospora cayetanensis* (*1*,*2*). Humans are the only known hosts of this parasite, which is transmissible by ingestion of fecally contaminated food or water. Direct person-to-person transmission is unlikely because the oocyst stage of the parasite that is shed in the feces of infected persons must undergo an extrinsic maturation process, thought to require at least 1–2 weeks in favorable environmental conditions, to become transmissible (*2*). *Cyclospora* oocysts are hardy; treatment of food or water with routine chemical disinfection or sanitizing methods is unlikely to kill the parasite.

*Cyclospora* infection can be asymptomatic or symptomatic, although asymptomatic infection is thought to be most common in settings where *Cyclospora* is endemic (*2*). Among persons who develop symptomatic *Cyclospora* infection, the incubation period averages 1 week (range: 2 to >14 days). Symptoms of cyclosporiasis can include watery diarrhea (most common), loss of appetite, weight loss, abdominal cramping, bloating, increased intestinal gas, nausea, fatigue, vomiting, and low-grade fever (*2*,*3*). The recommended treatment for cyclosporiasis is trimethoprim-sulfamethoxazole; no highly effective alternative treatments have been identified (*4*). If untreated, the illness can be prolonged, with remitting-relapsing symptoms for weeks to months, but ultimately self-limiting (*2*).

**FIGURE 1:**
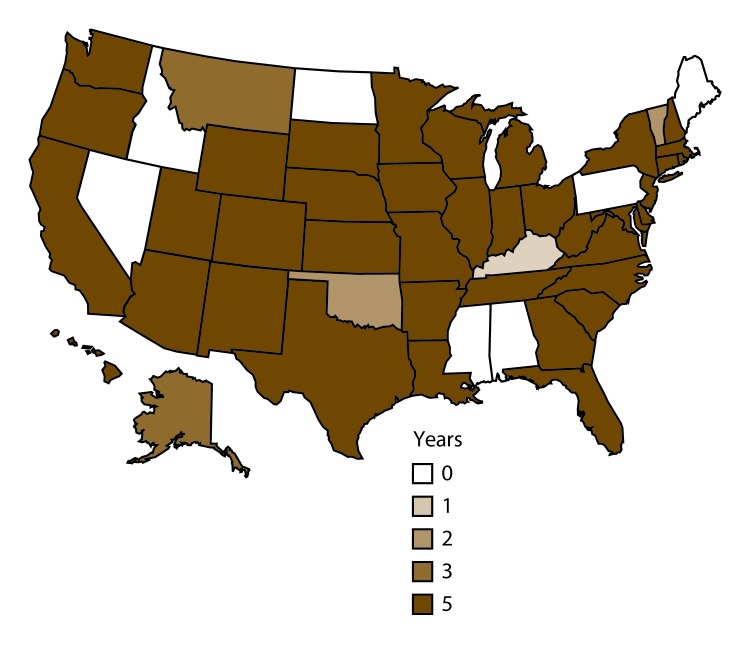
Number of years of reportability for cyclosporiasis, by public health jurisdiction — United States, 2011–2015* * Cyclosporiasis was a reportable condition in 39 jurisdictions (38 states and New York City) throughout the 5-year surveillance period. Cyclosporiasis was reportable in six jurisdictions during only part of the 5-year period (Alaska, 2013–2015; the District of Columbia, 2014–2015; Kentucky, 2015; Montana, 2013–2015; Oklahoma, 2011–2012; and Vermont, 2014–2015). Cyclosporiasis was not reportable in seven states (Alabama, Idaho, Maine, Mississippi, Nevada, North Dakota, and Pennsylvania) during any part of the 5-year period.

Cyclosporiasis is most common in tropical and subtropical regions of the world. In the United States, cases of cyclosporiasis have been reported among travelers to such areas (*1*,*2*,*5*). *Cyclospora* emerged as a foodborne pathogen of concern during the mid-1990s. In the context of large multistate outbreaks during 1996 and 1997 (*2*,*3*,*6*), cyclosporiasis became a nationally notifiable disease in January 1999 (*1*,*5*). As of 2015, cyclosporiasis was reportable in 44 U.S. public health jurisdictions (42 states, the District of Columbia, and New York City [NYC]) (Figure 1). Since the mid-1990s, U.S. foodborne outbreaks of cyclosporiasis have been detected almost every year, typically during spring and summer months. Various types of imported fresh produce (e.g., basil, raspberries, and snow peas) have been implicated as vehicles of infection (*2*,*3*,*6*–*10*). CDC, in collaboration with state and local public health authorities, investigates cases of cyclosporiasis for epidemiologic evidence of linkage to other cases. Validated molecular typing tools, which could facilitate detection and investigation of outbreaks, are not yet available for *C. cayetanensis*. 

This report summarizes national surveillance data for 2011–2015, including data for outbreak-associated cases and seemingly sporadic cases acquired in the United States, as well as international travel-associated cases. Public health authorities, health care providers, and laboratorians can use the findings summarized in this report to inform and improve detection, reporting, investigation, and prevention of cases and outbreaks of cyclosporiasis.

## Methods

### Definitions

Cases of cyclosporiasis were classified by health departments as confirmed or probable using the surveillance case definition approved by the Council of State and Territorial Epidemiologists in 2010 (Box). The year in which a patient’s case was counted was assigned by the health department and might reflect the year of symptom onset, diagnosis, or reporting to or by the health department. Cases were classified as travel associated or domestically acquired on the basis of the patient’s travel history during the pertinent exposure period (i.e., the 14 days before illness onset). If the patient reported having a history of international travel (i.e., travel outside the United States) during all or part of the exposure period, the patient’s case was classified as travel associated. Travel to a U.S. territory was classified as international. If the patient reported having no history of international travel, the patient’s case was classified as domestically acquired. An outbreak was defined as at least two epidemiologically linked cases (e.g., a cluster[s] of cases among persons linked to a social or other type of event, restaurant, grocery store, or community and/or cases linked to a particular food item), at least one of which must have been laboratory confirmed.

BOX National surveillance case definition for cyclosporiasis, 2010*

**Clinical description**

An illness of variable severity caused by the protozoan parasite *Cyclospora cayetanensis*. The most common symptom is watery diarrhea. Other common symptoms include loss of appetite, weight loss, abdominal cramping and bloating, nausea, body aches, and fatigue. Vomiting and low-grade fever also might be noted.

**Laboratory criteria for diagnosis**

Laboratory confirmed: detection of *Cyclospora* organisms or DNA in stool, intestinal fluid or aspirate, or intestinal biopsy specimens.

**Case classification**

Probable: a case that meets the clinical description and is epidemiologically linked to a confirmed case.

Confirmed: a case that meets the clinical description and at least one of the criteria for laboratory confirmation.

### Data Sources and Analysis

For the 2011–2015 surveillance period, U.S. public health jurisdictions in which cyclosporiasis was a reportable condition notified CDC of laboratory-confirmed and probable cases through the National Notifiable Diseases Surveillance System (NNDSS), which is a voluntary, passive surveillance system. Although cyclosporiasis is a nationally notifiable condition, notifying CDC of cases is voluntary even for jurisdictions in which cyclosporiasis is reportable by law. The number of jurisdictions in which cyclosporiasis was reportable ranged from 40 (2011 and 2012) to 44 (2015) (Figure 1). Selected demographic data, such as state and county of residence, age, race, and ethnicity, are reported through NNDSS. Supplementary data were obtained through the CDC cyclosporiasis case report form or the more extensive Cyclosporiasis National Hypothesis Generating Questionnaire (CNHGQ). Since 2014, the CNHGQ has been used to collect detailed exposure data (e.g., fresh produce consumption, points of service [such as restaurants and grocery stores], and events attended) from persons who became ill during May–August, the period during which most reported U.S. cases and outbreaks have occurred. CDC also encourages the use of the CNHGQ in lieu of the case report form during other times of the year (September–April), if warranted by the number and characteristics of reported domestically acquired cases.

Cyclosporiasis case data reported to CDC were compiled into an aggregate database. The data reported through various means (e.g., NNDSS, case report form, and CNHGQ) were reconciled and merged using case identification numbers and available demographic data. If discrepancies were noted (e.g., if the specified onset date differed), data from the more detailed or complete record were used. Certain data in this report (e.g., case counts for particular periods) might differ from data that were previously published because of differences in the timelines for finalizing data or in the analytic approaches (*11*). In this report, unless otherwise specified, cases were tallied by state or jurisdiction of residence of the infected person, which might differ from the location of exposure, and confirmed and probable cases were combined for further analyses. Outbreaks of cyclosporiasis were identified by public health authorities and reported to CDC.

Data analyses were conducted using SAS (version 9.4; SAS Institute). Differences among cases by age, sex, travel history, and hospitalization status were analyzed using two-sided t-tests and chi-square tests; a two-tailed p value of <0.05 was considered significant. Intercensal estimates of state/area populations by year, obtained from the U.S. Census Bureau (*12*), were used to calculate incidence rates per 100,000 persons. For calculations of total (versus jurisdiction-specific) annual incidence rates, the denominator comprised the sum of the populations of all of the states and jurisdictions in which cyclosporiasis was a reportable condition during the pertinent year.

## Results

For the 2011–2015 surveillance period, CDC was notified of 2,207 cases of cyclosporiasis: 1,988 confirmed cases (90.1%) and 219 probable cases (9.9%) (Table 1). These 2,207 cases were reported by 38 jurisdictions, including 37 states and NYC (Table 2). The annual number of jurisdictions that reported cases to CDC ranged from 18 in 2012 to 33 in 2015. The annual number of reported cases ranged from 130 in 2012 to 798 in 2013; the annual population-adjusted incidence rate ranged from 0.05 cases per 100,000 persons in 2012 to 0.29 in 2013 (Table 2).

**TABLE 1:** Number* and percentage^†^ of reported cases of cyclosporiasis, by selected characteristics — United States, 2011–2015

Characteristic	No. (%)
**Case classification** ^§^	
Confirmed	1,988 (90.1)
Probable	219 (9.9)
**Sex**	
Male	929 (42.1)
Female	1,160 (52.6)
Unknown/Missing	118 (5.3)
**Age group (yrs)**	
0–9	40 (1.8)
10–19	61 (2.8)
20–29	193 (8.7)
30–39	334 (15.1)
40–49	424 (19.2)
50–59	499 (22.6)
60–69	356 (16.1)
70–79	143 (6.5)
≥80	45 (2.0)
Unknown/Missing	112 (5.1)
**Race**	
American Indian/Alaska Native	0 (0)
Asian/Pacific Islander	18 (0.8)
Black	40 (1.8)
White	745 (33.8)
Other	14 (0.6)
Unknown/Missing	1,390 (63.0)
**Ethnicity**	
Hispanic	239 (10.8)
Non-Hispanic	1,174 (53.2)
Unknown/Missing	794 (36.0)
**Region** ^¶^	
New England	98 (4.4)
Mid-Atlantic	247 (11.2)
East North Central	174 (7.9)
West North Central	289 (13.1)
South Atlantic	351 (15.9)
East South Central	7 (0.3)
West South Central	973 (44.1)
Mountain	32 (1.4)
Pacific	36 (1.6)

* N = 2,207.

^†^ Percentages might not total 100% because of rounding.

^§^ Confirmed: a case that meets the clinical description and at least one of the criteria for laboratory confirmation. Probable: a case that meets the clinical description and is epidemiologically linked to a confirmed case. **Source**: CDC. Cyclosporiasis (*Cyclospora cayetanensis*) 2010 Case Definition. Atlanta, GA: US Department of Health and Human Services, CDC; 2017. https://wwwn.cdc.gov/nndss/conditions/cyclosporiasis/case-definition/2010/

^¶^ Cases were reported and totaled by state/area of residence, which might differ from location of exposure. Regions were defined as New England (Connecticut, Maine, Massachusetts, New Hampshire, Rhode Island, and Vermont), Mid-Atlantic (New Jersey, New York, New York City, and Pennsylvania), East North Central (Illinois, Indiana, Michigan, Ohio, and Wisconsin), West North Central (Iowa, Kansas, Minnesota, Missouri, Nebraska, North Dakota, and South Dakota), South Atlantic (Delaware, District of Columbia, Florida, Georgia, Maryland, North Carolina, South Carolina, Virginia, and West Virginia), East South Central (Alabama, Kentucky, Mississippi, and Tennessee), West South Central (Arkansas, Louisiana, Oklahoma, and Texas), Mountain (Arizona, Colorado, Idaho, Montana, Nevada, New Mexico, Utah, and Wyoming), and Pacific (Alaska, California, Hawaii, Oregon, and Washington).

**TABLE 2:** Number* and incidence rate^†^ of reported cases of cyclosporiasis, by state/area of residence^§^ and year^¶^ — United States, 2011–2015

State/Area	2011		2012		2013		2014		2015		Total
	No.	Rate	No.	Rate	No.	Rate	No.	Rate	No.	Rate	No.
Alabama	—**	—	—	—	—	—	—	—	—	—	**—**
Alaska	—	—	—	—	0	0	0	0	0	0	**0**
Arizona	0	0	0	0	0	0	2	0.03	1	0.01	**3**
Arkansas	0	0	0	0	17	0.57	1	0.03	3	0.10	**21**
California	2	0.01	1	<0.01	2	0.01	2	0.01	15	0.04	**22**
Colorado	0	0	1	0.02	1	0.02	1	0.02	8	0.15	**11**
Connecticut	10	0.28	6	0.17	3	0.08	8	0.22	16	0.45	**43**
Delaware	1	0.11	0	0	0	0	0	0	1	0.11	**2**
District of Columbia	—	—	—	—	—	—	0	0	0	0.00	**0**
Florida	61	0.32	25	0.13	47	0.24	33	0.17	32	0.16	**198**
Georgia	36	0.37	2	0.02	6	0.06	6	0.06	34	0.33	**84**
Hawaii	0	0	0	0	0	0	0	0	0	0	**0**
Idaho	—	—	—	—	—	—	—	—	—	—	**—**
Illinois	1	0	2	0.02	23	0.18	14	0.11	21	0.16	**61**
Indiana	26	0	0	0	1	0.02	2	0.03	0	0	**29**
Iowa	1	0.03	0	0	148	4.79	0	0	4	0.13	**153**
Kansas	0	0	0	0	4	0.14	1	0.03	6	0.21	**11**
Kentucky	—	—	—	—	—	—	—	—	0	0	**0**
Louisiana	1	0.02	1	0.02	3	0.06	0	0	1	0.02	**6**
Maine	—	—	—	—	—	—	—	—	—	—	**—**
Maryland	2	0.03	4	0.07	0	0	4	0.07	3	0.05	**13**
Massachusetts	6	0.09	1	0.02	5	0.07	18	0.27	21	0.31	**51**
Michigan	8	0.08	0	0	2	0.02	8	0.08	8	0.08	**26**
Minnesota	2	0	0	0	3	0.06	5	0.09	1	0.02	**11**
Mississippi	—	—	—	—	—	—	—	—	—	—	**—**
Missouri	1	0.02	2	0.03	5	0.08	1	0.02	5	0.08	**14**
Montana	—	—	—	—	0	0	2	0.20	3	0.29	**5**
Nebraska	1	0.05	0	0	91	4.87	3	0.16	4	0.21	**99**
Nevada	—	—	—	—	—	—	—	—	—	—	**—**
New Hampshire	0	0	0	0	1	0.08	0	0	3	0.23	**4**
New Jersey	9	0.10	7	0.08	13	0.15	16	0.18	21	0.23	**66**
New Mexico	1	0.05	0	0	0	0	0	0	2	0.10	**3**
New York^††^	30	0.15	21	0.11	21	0.11	37	0.19	72	0.36	**181**
North Carolina	1	0.01	2	0.02	0	0	4	0.04	4	0.04	**11**
North Dakota	—	—	—	—	—	—	—	—	—	—	**—**
Ohio	7	0.06	0	0	7	0.06	1	0.01	2	0.02	**17**
Oklahoma	0	0	0	0	—	—	—	—	—	—	**0**
Oregon	0	0	1	0.03	0	0	1	0.03	0	0	**2**
Pennsylvania	—	—	—	—	—	—	—	—	—	—	**—**
Rhode Island	0	0	0	0	0	0	0	0	0	0	**0**
South Carolina	1	0.02	0	0	0	0	18	0.37	2	0.04	**21**
South Dakota	0	0	0	0	1	0.12	0	0	0	0	**1**
Tennessee	2	0.03	2	0.03	1	0.02	1	0.02	1	0.02	**7**
Texas	18	0.07	51	0.20	362	1.37	200	0.74	315	1.15	**946**
Utah	0	0	0	0	0	0	1	0.03	8	0.27	**9**
Vermont	—	—	—	—	—	—	0	0	0	0	**0**
Virginia	4	0.05	1	0.01	4	0.05	4	0.05	8	0.10	**21**
Washington	5	0.07	0	0	0	0	2	0.03	5	0.07	**12**
West Virginia	0	0	0	0	0	0	1	0.05	0	0	**1**
Wisconsin	0	0	0	0	26	0.45	1	0.02	14	0.24	**41**
Wyoming	0	0	0	0	1	0.17	0	0	0	0	**1**
**Total**	**237^§§^**	**0.09^¶¶^**	**130**	**0.05**	**798**	**0.29**	**398**	**0.14**	**644**	**0.22**	**2,207**

* N = 2,207.

^†^ Incidence rate per 100,000 persons on the basis of U.S. intercensal state/area population estimates by year. **Source**: US Census Bureau. Annual estimates of the resident population: April 1, 2010 to July 1, 2017. Washington, DC: US Census Bureau; 2018. https://factfinder.census.gov/faces/tableservices/jsf/pages/productview.xhtml?pid=PEP_2017_PEPANNRES&src=pt

^§^ Cases were reported by state/area of residence, which might differ from location of exposure. For the 2011–2015 surveillance period (year as reported by health department), CDC was notified of a total of 2,207 cases by 38 jurisdictions (37 states and New York City). Jurisdictions (50 states and the District of Columbia) are listed regardless of whether they notified CDC of any cases or whether cyclosporiasis was a reportable condition. Cases reported by New York State and New York City were totaled together for the purpose of the incidence calculations.

^¶^ Year as reported by health department.

** Not reportable.

^††^ Including New York City, which accounted for 124 (68.5%) of New York State’s 181 reported cases. New York City reported cases for all 5 surveillance years (19 for 2011, 16 for 2012, 15 for 2013, 23 for 2014, and 51 for 2015).

^§§^ Of the 237 total cases counted for 2011, 85 were probable cases associated with an outbreak in Georgia that were not reported in the National Notifiable Diseases Surveillance System (or included in case counts previously published [**Source**: CDC. MMWR: Summary of notifiable infectious diseases*.* Atlanta, GA: US Department of Health and Human Services, CDC; 2019. https://www.cdc.gov/mmwr/mmwr_nd/index.html]), which occurred among residents of 14 states, including the state of exposure. Ten additional probable cases associated with the same outbreak were not counted here: nine of these 10 occurred among residents of states in which cyclosporiasis was not a reportable condition, and the state of residence of the other patient was not known.

^¶¶^ For calculations of total annual incidence rates, the denominator comprised the sum of the populations of all of the states/areas in which cyclosporiasis was a reportable condition during the pertinent year.

Of the 2,207 total reported cases, 1,577 (71.5%) were among residents of five states: Texas (946 cases), Florida (198 cases), New York (181 cases, including 124 in NYC), Iowa (153 cases), and Nebraska (99 cases). More than 50 cases (range: 51−84) were reported by each of four other states: Georgia, New Jersey, Illinois, and Massachusetts. Overall, 12 (31.6%) of the 38 jurisdictions that reported at least one case reported <10 total cases.

Among the 2,207 patients, 52.6% were female, 42.1% were male, and the sex was unknown for 5.3% (Table 1). The median age was 49 years (range: 1–95 years; interquartile range [IQR]: 36–60 years). A total of 73.1% of the reported cases were among persons aged 30–69 years, with at least 15% of the cases in each of four consecutive 10-year age groups (i.e., 30–39, 40–49, 50–59, and 60–69 years); <5% of reported cases were among persons aged <20 years (Table 1). Mean patient age was not significantly different for males compared with females. Among the 1,526 patients with available information, 103 (6.7%) reported having been hospitalized. Patients who had been hospitalized were significantly older than those who had not been hospitalized. No deaths were reported.

**FIGURE 2:**
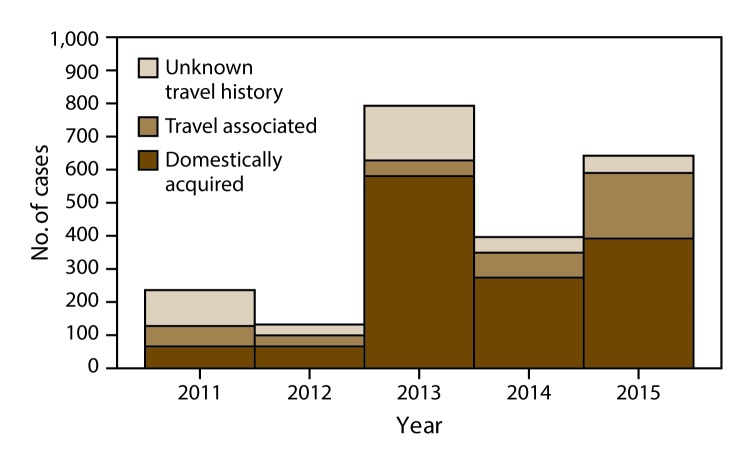
Number* of reported cases of cyclosporiasis, by year^†^ and international travel history^§^ — United States, 2011–2015 * N = 2,207. ^†^ Year as reported by health department. ^§^ Cases were classified as travel associated or domestically acquired on the basis of the patient’s travel history during the pertinent exposure period (i.e., the 14 days before illness onset). If the patient reported having a history of international travel (i.e., travel outside the United States) during all or part of the exposure period, the patient’s case was classified as travel associated. If the patient reported having no history of international travel, the patient’s case was classified as domestically acquired.

Overall, 1,384 (62.7%) of the reported cases were classified as domestically acquired, and 415 (18.8%) were classified as travel associated; the travel status was unknown for 408 patients (18.5%) (Tables 3 and 4). The annual proportion of reported cases with missing or unknown travel history decreased from 46.8% in 2011 to 7.8% in 2015 (Table 4) (Figure 2). Persons with domestically acquired infection were significantly older than persons with travel-associated illness; however, classification by travel history was not significantly different for males compared with females.

**TABLE 3:** Number* and percentage^†^ of reported cases of cyclosporiasis, by state/area of residence^§^ and international travel history^¶^ — United States, 2011–2015

State/Area (No. of yrs condition was reportable)**	Domestically acquired	Travel associated	Unknown travel history	Total
	No. (%)	No. (%)	No. (%)	No. (%)
Alabama (0)	—^††^	—	—	**—**
Alaska (3)	0 (0)	0 (0)	0 (0)	**0 (0)**
Arizona	2 (0.1)	1 (0.2)	0 (0)	**3 (0.1)**
Arkansas	15 (1.1)	0 (0)	6 (1.5)	**21 (1.0)**
California	6 (0.4)	11 (2.7)	5 (1.2)	**22 (1.0)**
Colorado	1 (0.1)	7 (1.7)	3 (0.7)	**11 (0.5)**
Connecticut	19 (1.4)	21 (5.1)	3 (0.7)	**43 (1.9)**
Delaware	0 (0)	0 (0)	2 (0.5)	**2 (0.1)**
District of Columbia (2)	0 (0)	0 (0)	0 (0)	**0 (0)**
Florida	127 (9.2)	44 (10.6)	27 (6.6)	**198 (9.0)**
Georgia	42 (3.0)	10 (2.4)	32 (7.8)	**84 (3.8)**
Hawaii	0 (0)	0 (0)	0 (0)	**0 (0)**
Idaho (0)	—	—	—	**—**
Illinois	36 (2.6)	12 (2.9)	13 (3.2)	**61 (2.8)**
Indiana	2 (0.1)	0 (0)	27 (6.6)	**29 (1.3)**
Iowa	137 (9.9)	4 (1.0)	12 (2.9)	**153 (6.9)**
Kansas	5 (0.4)	2 (0.5)	4 (1.0)	**11 (0.5)**
Kentucky (1)	0 (0)	0 (0)	0 (0)	**0 (0)**
Louisiana	5 (0.4)	1 (0.2)	0 (0)	**6 (0.3)**
Maine (0)	—	—	—	**—**
Maryland	5 (0.4)	6 (1.4)	2 (0.5)	**13 (0.6)**
Massachusetts	29 (2.1)	9 (2.2)	13 (3.2)	**51 (2.3)**
Michigan	9 (0.7)	2 (0.5)	15 (3.7)	**26 (1.2)**
Minnesota	7 (0.5)	2 (0.5)	2 (0.5)	**11 (0.5)**
Mississippi (0)	—	—	—	**—**
Missouri	7 (0.5)	5 (1.2)	2 (0.5)	**14 (0.6)**
Montana (3)	5 (0.4)	0 (0)	0 (0)	**5 (0.2)**
Nebraska	53 (3.8)	5 (1.2)	41 (10.0)	**99 (4.5)**
Nevada (0)	—	—	—	**—**
New Hampshire	4 (0.3)	0 (0)	0 (0)	**4 (0.2)**
New Jersey	26 (1.9)	31 (7.5)	9 (2.2)	**66 (3.0)**
New Mexico	2 (0.1)	1 (0.2)	0 (0)	**3 (0.1)**
New York State^§§^	25 (1.8)	23 (5.5)	9 (2.2)	**57 (2.6)**
New York City	54 (3.9)	58 (14.0)	12 (2.9)	**124 (5.6)**
North Carolina	1 (0.1)	2 (0.5)	8 (2.0)	**11 (0.5)**
North Dakota (0)	—	—	—	**—**
Ohio	5 (0.4)	2 (0.5)	10 (2.5)	**17 (0.8)**
Oklahoma (2)	0 (0)	0 (0)	0 (0)	**0 (0)**
Oregon	0 (0)	2 (0.5)	0 (0)	**2 (0.1)**
Pennsylvania (0)	—	—	—	**—**
Rhode Island	0 (0)	0 (0)	0 (0)	**0 (0)**
South Carolina	15 (1.1)	1 (0.2)	5 (1.2)	**21 (1.0)**
South Dakota	1 (0.1)	0 (0)	0 (0)	**1 (0)**
Tennessee	1 (0.1)	4 (1.0)	2 (0.5)	**7 (0.3)**
Texas	684 (49.4)	130 (31.3)	132 (32.4)	**946 (42.9)**
Utah	3 (0.2)	6 (1.4)	0 (0)	**9 (0.4)**
Vermont (2)	0 (0)	0 (0)	0 (0)	**0 (0)**
Virginia	16 (1.2)	3 (0.7)	2 (0.5)	**21 (1.0)**
Washington	3 (0.2)	8 (1.9)	1 (0.2)	**12 (0.5)**
West Virginia	1 (0.1)	0 (0)	0 (0)	**1 (0)**
Wisconsin	30 (2.2)	2 (0.5)	9 (2.2)	**41 (1.9)**
Wyoming	1 (0.1)	0 (0)	0 (0)	**1 (0)**
**Total**	**1,384 (100.0)**	**415 (100.0)**	**408 (100.0)**	**2,207 (100.0)**

* N = 2,207.

^†^ Percentages might not total 100% because of rounding.

^§^ Cases were reported by state/area of residence, which might differ from location of exposure. For the 2011–2015 surveillance period (year as reported by health department), CDC was notified of a total of 2,207 cases by 38 jurisdictions (37 states and New York City). Jurisdictions (50 states, the District of Columbia, and New York City) are listed regardless of whether they notified CDC of any cases or whether cyclosporiasis was a reportable condition.

^¶^ Cases were classified as travel associated or domestically acquired on the basis of the patient's travel history during the pertinent exposure period (i.e., the 14 days before illness onset). If the patient reported having a history of international travel (i.e., travel outside the United States) during all or part of the exposure period, the patient's case was classified as travel associated. If the patient reported having no history of international travel, the patient's case was classified as domestically acquired.

** The number of years that cyclosporiasis was a reportable condition in the listed jurisdiction if <5 years.

^††^ Not reportable.

^§§^ New York State exclusive of New York City.

**TABLE 4:** Number* and percentage of reported cases of cyclosporiasis, by year^†^ and international travel history^§^ — United States, 2011–2015

Year(s)	Domestically acquired	Travel associated	Unknown travel history	Total
	No. (%)^¶^	No. (%)	No. (%)	No. (%)
2011	66 (27.8)	60 (25.3)	111 (46.8)	**237 (100.0)**
2012	64 (49.2)	34 (26.2)	32 (24.6)	**130 (100.0)**
2013	585 (73.3)	44 (5.5)	169 (21.2)	**798 (100.0)**
2014	275 (69.1)	77 (19.3)	46 (11.6)	**398 (100.0)**
2015	394 (61.2)	200 (31.1)	50 (7.8)	**644 (100.0)**
**2011–2015**	**1,384 (62.7)**	**415 (18.8)**	**408 (18.5)**	**2,207 (100.0)**

* N = 2,207.

^†^ Year as reported by health department.

^§^ Cases were classified as travel associated or domestically acquired on the basis of the patient's travel history during the pertinent exposure period (i.e., the 14 days before illness onset). If the patient reported having a history of international travel (i.e., travel outside the United States) during all or part of the exposure period, the patient's case was classified as travel associated. If the patient reported having no history of international travel, the patient's case was classified as domestically acquired.

^¶^ Percentages (per year[s]) in a row might not total 100% because of rounding.

### Domestically Acquired Cases

The 1,384 domestically acquired cases were reported by 36 jurisdictions (35 states and NYC), which were not always the same as the jurisdictions of exposure (Table 3). A median of 275 such cases were reported per year (range: 64–585) (Table 4). Of these 1,384 cases, 1,188 (85.8%) were reported by eight states: Texas (684 cases), Iowa (137 cases), Florida (127 cases), New York (79 cases, including 54 in NYC), Nebraska (53 cases), Georgia (42 cases), Illinois (36 cases), and Wisconsin (30 cases) (Table 3).

**FIGURE 3:**
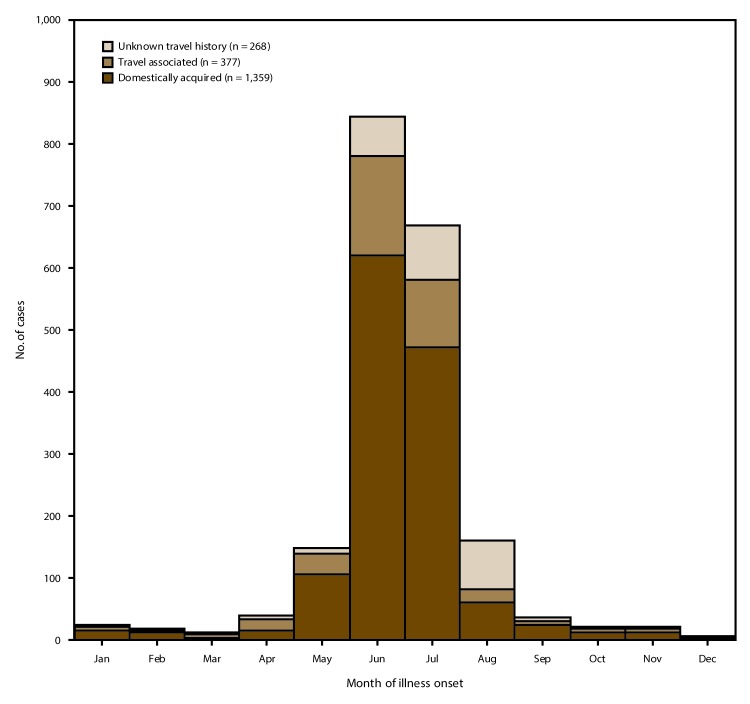
Number* of reported cases of cyclosporiasis, by month of illness onset^†^ and international travel history^§^ — United States, 2011–2015 * N = 2,004. ^†^ Data regarding month of illness onset were available for most (90.8%, or 2,004 of 2,207) of the patients whose cases were reported to CDC (2011, 205 of 237; 2012, 112 of 130; 2013, 738 of 798; 2014, 356 of 398; and 2015, 593 of 644). ^§^ Cases were classified as travel associated or domestically acquired on the basis of the patient’s travel history during the pertinent exposure period (i.e., the 14 days before illness onset). If the patient reported having a history of international travel (i.e., travel outside the United States) during all or part of the exposure period, the patient’s case was classified as travel associated. If the patient reported having no history of international travel, the patient’s case was classified as domestically acquired.

Domestically acquired cases occurred among persons with illness onset during every month except December; 1,095 (80.6%) of 1,359 cases with available information occurred in June (621 cases) or July (474 cases) (Figure 3). During 2011, illness onset peaked in July; in each of the other 4 surveillance years (2012–2015), illness onset peaked in June. The median age among persons with domestically acquired illness was 51 years (range: 1–95 years; IQR: 38–61 years).

### Travel-Associated Cases

The 415 travel-associated cases were reported by 30 jurisdictions (29 states and NYC); eight jurisdictions reported >10 cases (range: 11−130) among persons with a history of international travel (Table 3). The annual proportion of reported cases that were travel associated ranged from 5.5% in 2013 to 31.1% in 2015 (Table 4) (Figure 2). The cases occurred in all 12 months, with the highest numbers in June and July (163 and 108 cases, respectively) (Figure 3). The median age among persons with travel-associated illness was 45 years (range: 1–84 years; IQR: 34–56 years).

Among the 415 patients whose cases were classified as travel associated, the case records for 299 patients (72.0%) included the country or region of travel; 18 patients reported having traveled to more than one country (Table 5). Among the 327 country visits for which geographic information was reported, 287 visits (87.8%) were in the Americas, including Mexico (208 cases), the Caribbean (43 cases), Central America (31 cases), and South America (five cases). Forty visits (12.2%) were elsewhere in the world, including Asia (29 cases), Europe (nine cases), and Africa (two cases).

**TABLE 5:** Number of country visits for U.S. patients with travel-associated cases of cyclosporiasis and estimated percentages of overall U.S. outbound travel, by international destinations — United States, 2011–2015

Destination*	Travel-associated cases with known travel destination(s)		U.S. outbound travel data^¶^		
	Country visits	Also traveled elsewhere^§^			
	No. (%)^†^	No.	Estimated % of all travel	Period with available data	
Mexico	208 (63.6)	6	36.0	2011–2015	
Guatemala	16 (4.9)	1	—**	—	
Dominican Republic	15 (4.6)	0	3.8	2012, 2014–2015	
Cuba	9 (2.8)	1	—	—	
Costa Rica	5 (1.5)	2	1.3	2011, 2014–2015	
Haiti	5 (1.5)	1	—	—	
India	5 (1.5)	0	1.6	2011–2015	
Bahamas	4 (1.2)	1	1.6	2014–2015	
China	4 (1.2)	2	1.8	2011–2015	
Nicaragua	4 (1.2)	0	—	—	
Puerto Rico	4 (1.2)	3	—	—	
Other^††^	48 (14.7)	—	—	—	
**Total**	**327^††^ (100.0)**	**—**	**—**	**—**	

* Among the 415 patients with travel-associated cases, some information about the destination of travel was known for 299 (72.0%). These 299 patients reportedly had a total of 327 country visits during the 14-day exposure period. For the purpose of these analyses, travel to a U.S. territory was classified as international. Destinations are listed in descending order, by the number of country visits.

^†^ Percentages in this column do not total 100% because of rounding.

^§^ Among the 299 patients, 18 (6.0%) reported travel to more than one country: eight patients had traveled to two countries, and 10 patients had traveled to three.

^¶^ U.S. outbound travel estimates are based on data from the U.S. Department of Commerce, International Trade Administration, National Travel and Tourism Office (**Source**: National Travel and Tourism Office. 2015 U.S. resident travel abroad. Washington, DC: US Department of Commerce, International Trade Administration, National Travel and Tourism Office; 2016. https://travel.trade.gov/outreachpages/download_data_table/2015_US_Travel_Abroad.pdf). Travel estimates were unavailable if the sample size for a particular country/year was <400 respondents. The country-specific percentages were calculated by summing the number of country visits and dividing by the outbound visits to that country during the period(s) with available data.

** Not available or applicable.

^††^ Each of the specified destinations (or regions) of travel in the “other” category was associated with fewer than three patients. The destinations include 28 countries not listed. For two of the country visits, only region-level data were available (i.e., the Caribbean and Scandinavia). Overall, 287 (87.8%) of the 327 visits with at least region-level data were in the Americas, including Mexico (n = 208), the Caribbean (n = 43), Central America (n = 31), and South America (n = 5). The other 40 visits (12.2%) were elsewhere in the world, including Asia (n = 29), Europe (n = 9), and Africa (n = 2).

### Outbreaks

During 2011–2015, a total of 10 outbreaks of cyclosporiasis were investigated (*7*), which in aggregate were linked to 438 (19.8%) of the 2,207 total cases counted for this surveillance period. A median of two outbreaks were reported per year, with a median of 21 reported cases per outbreak (range: eight to 162). The persons with reported cases linked to these outbreaks became ill during May–August. A food vehicle of infection (i.e., a food item or ingredient thereof) was identified (or suspected) for at least five of the 10 outbreaks; the vehicles included a berry salad (suspected for one outbreak), cilantro (identified for at least three outbreaks), and a prepackaged salad mix (identified for one outbreak). The 10 outbreaks are summarized by year of occurrence. In the outbreak summaries, cases are classified by the state where the patients were likely exposed, which might have differed from their state of residence.

#### 2011

- **Florida**: Twelve cases (three confirmed and nine probable) were identified among four groups of persons who had eaten at the same Mexican-style restaurant and became ill during June and July (*13*). Although an analytic epidemiologic study was not conducted, all 12 patients had eaten food items that contained fresh cilantro and onions. The lack of records at the restaurant, distributor, and vendor precluded traceback investigations.
- **Georgia**: An outbreak associated with two events held at and catered by the same facility during July was investigated (*14*). Eighty-eight cases (three confirmed and 85 probable) among residents of 14 states who became ill during July and August were counted in this report; 10 additional probable cases associated with the outbreak were not counted because nine were among residents of states in which cyclosporiasis was not a reportable condition and one was in a patient whose state of residence was unknown (Table 2). In epidemiologic investigations, no single food item or ingredient thereof was significantly associated with illness.

#### 2012

- **Texas**: Sixteen cases (nine confirmed and seven probable) were identified among persons who had eaten at the same Mexican-style restaurant during June and July. On the basis of the results of a case-control study, fish tacos were the likely vehicle of infection (*13*); however, cilantro and cabbage, the two fresh-produce ingredients, were served mixed together and could not be distinguished in the epidemiologic investigation. After cilantro imported from Puebla, Mexico, was implicated for the outbreak of cyclosporiasis in Texas in 2013 (see following outbreak summary), an informational (nonregulatory) traceback investigation was conducted for the cilantro at the pertinent restaurant in Texas in 2012 and demonstrated that firms in Puebla, Mexico, were among the potential sources of the cilantro (*15*).

#### 2013

- **Iowa, Nebraska, and neighboring states**: In a multistate outbreak investigation in which a prepackaged salad mix was implicated, 162 laboratory-confirmed cases were identified among persons who had eaten salad at various locations of two related chain restaurants and became ill during June and July (*16*). The state of exposure was Iowa or Nebraska for all but five patients, including four exposed in Wisconsin and one exposed in South Dakota (*16*). The salad mix, which contained romaine and iceberg lettuce, red cabbage, and carrots, had been imported from Guanajuato, Mexico (*13*,*17*,*18*).
- **Texas**: Four clusters of cases (38 reported cases) were investigated among persons who became ill during June and July: three clusters were each associated with a different Mexican-style restaurant, and one was associated with a grocery store chain (*13*). In a case-control study of the largest restaurant-associated cluster (25 cases), fresh cilantro was the food ingredient most strongly associated with illness (*13*). All of the patients associated with the other three clusters also reported exposure to cilantro. Traceback investigations for the cilantro at the four cluster-associated establishments converged on three suppliers in Puebla, Mexico (*13*).
- **Wisconsin**: Eight cases (six confirmed and two probable) were identified among persons who had attended a potluck and became ill in July. A berry salad was the suspected vehicle of infection.

#### 2014

- **Michigan**: Fourteen cases (nine confirmed and five probable) were identified among persons associated with two events held at a conference center who became ill during June and July. In epidemiologic investigations, no food item or ingredient thereof was identified as significantly associated with illness.
- **South Carolina**: A temporospatial cluster of 13 cases (three confirmed and 10 probable) was identified among residents of a gated community who became ill in July. No food vehicle of infection was identified.
- **Texas**: Multiple clusters were investigated, including five clusters that each was associated with a different Mexican-style restaurant and that in aggregate were linked to 26 reported cases (13 confirmed and 13 probable) among persons who became ill during June and July (*19*). In epidemiologic investigations, fresh cilantro was identified as the vehicle of infection; traceback investigations for the cilantro converged on multiple firms in Puebla, Mexico.

#### 2015

- **Georgia, Texas, and Wisconsin**: Fresh cilantro imported from Mexico was identified as the vehicle of infection for a multistate outbreak with 61 reported cases (56 confirmed and five probable) associated with unrelated Mexican-style restaurants in Texas (35 cases), Georgia (18 cases), and Wisconsin (eight cases) among persons who became ill during May–July. An additional 29 probable cases associated with the restaurant in Texas were not counted because individual case data were not reported.

## Discussion

Cyclosporiasis is an ongoing U.S. public health concern, with most of the reported cases occurring during spring and summer months. For the 2011–2015 surveillance period, the numbers and incidence of reported cases fluctuated from year to year. Overall, the majority (62.7%) of reported cases were among persons without a history of international travel during the 14-day exposure period. *Cyclospora* is not thought to be endemic in the United States, although the possibility of low-level endemicity of the parasite cannot be excluded (*2*). Foodborne outbreaks of cyclosporiasis have been identified in the United States almost every year since the mid-1990s. For the subset of U.S. outbreaks documented from the mid-1990s through 2015 for which both the food vehicle of infection and its source were identified, the implicated produce item had been imported. The applicable vehicle-source pairs before this surveillance period included raspberries from Guatemala (1996, 1997, and 2000), snow peas from Guatemala (2004), mesclun lettuce from Peru (1997), and basil from Peru (2005) (*2*,*3*,*6*,*7*,*9*,*10*). During 2011–2015, the identified vehicle-source pairs were a prepackaged salad mix from Mexico (2013) and cilantro from Mexico (2013, 2014, and 2015 and, potentially, 2011 and 2012) (*7*,*13*,*15*–*18*).

The domestically acquired cases reported for the 2011–2015 surveillance period were dispersed geographically. However, three states reported the majority (68.5%) of the domestically acquired cases: Texas, Iowa, and Florida. Florida reported the highest number of total cases for 2011. For the 2012–2015 surveillance period, Texas reported the highest number of total cases. Cases reported among persons from the East-South Central, Mountain, and Pacific regions accounted for only 3.4% of total cases. The extent to which the geographic distribution of cases reflects differences in exposure or infection rates or in laboratory or surveillance practices is unknown.

Overall, at least 18.8% of cases occurred among persons with a history of international travel; the majority of persons with available information had visited Mexico (63.6%), the Caribbean (13.1%), and/or Central America (9.5%). Mexico was the most frequent destination for patients with travel-associated cases for whom information was available and for U.S. international travelers overall (Table 5) (*20*). However, whereas Mexico accounted for 63.6% of the country visits among patients with travel-associated cases, the estimated annual proportion of U.S. international travelers who went to Mexico was 36.0% (averaged over the 5-year period) (*20*). Such comparisons could not be made for the Caribbean and Central America because U.S. outbound travel data for this period were not available for all of the pertinent Caribbean and Central American countries (*20*). The large proportion of travel-associated cases that occurred among travelers to Latin America is consistent with previous surveillance reports (*1*,*5*) and with U.S. outbreaks associated with fresh produce imported from this region. Because many of the persons who were classified as having travel-associated cases had spent at least 1 day during their 14-day exposure period in the United States (and some persons had traveled to more than one country), the country in which they became infected could not be definitively established.

Reported cases of cyclosporiasis primarily occurred among adults, with relatively few cases among children and older adults. The extent to which the relatively few reported cases among children reflects lower rates of exposure (e.g., because of food preferences), of infection or illness, or of diagnosis or reporting of cases is unknown.

At least one outbreak was identified each year during 2011–2015. A total of 10 outbreaks were investigated, two of which were found to be associated with exposures in more than one state: a multistate outbreak in Iowa, Nebraska, and neighboring states in 2013 (*16*); and a multistate outbreak in Georgia, Texas, and Wisconsin in 2015. Although epidemiologic investigations were conducted, a food vehicle of infection was identified for only five outbreaks (50%), if the outbreaks in Florida in 2011 (*13*) and in Texas in 2012 (*13*), which might have been harbingers of the outbreaks linked to cilantro during 2013–2015, are not counted. Cilantro was the only produce item identified as a vehicle of infection during multiple years in the surveillance period.

Identifying a food vehicle of *Cyclospora* infection can be challenging, in part because fresh produce is often served mixed together with other types of produce (e.g., in a salad) or in inconspicuous ways (e.g., as a garnish). In addition, because fresh produce items typically have a short shelf life, vehicles implicated on the basis of epidemiologic data usually are not available for laboratory testing by the time they are identified in outbreak investigations. The occurrence of multiple distinct outbreaks associated with different food vehicles of infection during the same time frame, as was documented in 2013 (*7*,*17*), also can complicate outbreak investigations.

Although only approximately 20% of the reported cases were directly linked to U.S. outbreaks, many of the seemingly sporadic cases among persons without a history of international travel might have been associated with identified or unidentified outbreaks; however, those potential associations were not detected using the available epidemiologic information. The challenges inherent to linking cases of cyclosporiasis to each other, especially if the cases are not associated with the same event or point of service (e.g., restaurant), are compounded by the lack of validated molecular typing tools for *C. cayetanensis*. As part of CDC’s Advanced Molecular Detection program, CDC scientists are working to develop and validate typing methods for this eukaryotic pathogen. To aid in the development and validation of such typing tools, public health partners are encouraged to submit stool specimens from persons with confirmed *Cyclospora* infection to CDC. In the future, molecular typing tools could facilitate outbreak detection, investigation, and prevention, by helping to link cases to each other, to food vehicles of infection, and to the sources of the vehicles.

## Limitations

The findings in this report are subject to at least three limitations. First, underdiagnosis of cases is likely, even of cases among persons with diarrheal illness who seek medical attention and who provide stool specimens for testing. Routine ova and parasite examinations of stool typically do not detect *Cyclospora* oocysts, unless health care providers explicitly request testing for this parasite. Gastrointestinal polymerase chain reaction panels are increasingly being used for diagnostic testing for patients with diarrheal illness and have the potential to increase case detection. However, as of April 2019, only one of the several commercially available multiplex gastrointestinal panels (a panel that became available in 2014) includes a target for *Cyclospora*. Therefore, if indicated, testing for *Cyclospora* still should be explicitly requested. Second, even if cases of cyclosporiasis are diagnosed, underreporting of cases is likely, in part because NNDSS is a passive surveillance system, cyclosporiasis is not a reportable condition in all U.S. public health jurisdictions, and notifying CDC of cases is voluntary even for jurisdictions in which cyclosporiasis is a reportable condition. Finally, even if cases are reported, the information provided to CDC might be limited in scope, quality, or timeliness.

For some of the reported cases, pertinent information was missing or unavailable (e.g., the patient’s illness onset date or international travel history), as were the detailed exposure data relevant to detection and investigation of outbreaks. Timely submission of complete and accurate data is especially important in the context of potential or documented outbreaks to inform investigations and control measures and thereby to stop ongoing transmission and to prevent future outbreaks. However, the relatively long incubation period for cyclosporiasis (i.e., a biologic delay that averages 1 week) is compounded by system delays in detecting and reporting cases and interviewing patients. On the basis of data provided to CDC, the median intervals from illness onset to diagnosis and from diagnosis to initial notification of CDC were 20 and 29 days, respectively. Although CDC typically did not have information regarding when cases were reported to health departments, patient interviews were often conducted a month or longer after onset of illness. Such delays could impair patients’ recall of their food exposures and thereby decrease the likelihood that food vehicles of infection are identified and that evidence-based intervention and prevention measures are implemented.

## Conclusion

Travelers to areas of known endemicity (e.g., in the tropics and subtropics) should follow food and water precautions similar to those for other enteric pathogens but should be advised that use of routine chemical disinfection or sanitizing methods is unlikely to kill *C. cayetanensis*. Health care providers should consider testing for *Cyclospora* in persons with persistent or remitting-relapsing diarrheal illness, especially for persons with a history of travel to areas of known endemicity or with symptom onset during spring or summer. Laboratory testing for *Cyclospora* should be explicitly requested because such testing is not typically part of routine examinations for ova and parasites and is not included in all multiplex gastrointestinal panels. Cases of cyclosporiasis should be promptly reported to state or local public health authorities, who are encouraged to notify CDC of the cases. During periods and seasons when increased numbers of domestically acquired cases are reported, the CNHGQ should be used to facilitate outbreak detection and hypothesis generation. Thorough investigations can lead to evidence-based public health action (e.g., the regional and seasonal import alert the U.S. Food and Drug Administration issued in August 2015 for fresh cilantro from Puebla, Mexico [April–August of each year]) (*15*). National surveillance and research efforts to develop and validate molecular typing tools for *C. cayetanensis* should remain U.S. public health priorities (*21*).

## Footnotes

* **Source**: CDC. Cyclosporiasis (*Cyclospora cayetanensis*) 2010 Case Definition. Atlanta, GA: US Department of Health and Human Services, CDC; 2017. https://wwwn.cdc.gov/nndss/conditions/cyclosporiasis/case-definition/2010/
